# Serum Alkaline Phosphatase Levels in Pediatric Kikuchi‐Fujimoto Disease: A Retrospective Observational Analysis

**DOI:** 10.1002/iid3.70129

**Published:** 2025-01-21

**Authors:** Shintaro Fujiwara, Yousuke Higuchi, Junya Shimizu

**Affiliations:** ^1^ Department of Pediatrics NHO Okayama Medical Center Okayama Japan

**Keywords:** histiocytic necrotizing lymphadenitis, infectious mononucleosis, Kikuchi‐Fujimoto disease, serum alkaline phosphatase

## Abstract

**Aim:**

Kikuchi‐Fujimoto disease (KFD) rarely affects pediatric patients and is characterized by prolonged fever and cervical lymphadenopathy. The diagnosis of KFD remains challenging and often requires an invasive biopsy. Low serum alkaline phosphatase levels have frequently been observed in patients with KFD; however, the clinical significance of low serum alkaline phosphatase levels remains unclear.

**Methods:**

This retrospective study included pediatric patients aged < 16 years who were pathologically or clinically diagnosed with KFD and infectious mononucleosis between April 2016 and March 2023. Serum alkaline phosphatase levels were analyzed employing age‐ and sex‐specific reference intervals. Clinical and laboratory data were evaluated to determine their association with serum alkaline phosphatase levels.

**Results:**

Thirty patients with KFD and 23 patients with infectious mononucleosis were included in the study. Seventeen patients with KFD (56.7%) had serum alkaline phosphatase levels below the 2.5th percentile of the age‐ and sex‐specific reference intervals. Serum alkaline phosphatase levels were significantly lower in patients with KFD than in those with infectious mononucleosis. Clinical and other laboratory findings were not significantly different between patients with KFD with or without a decline in serum alkaline phosphatase levels.

**Conclusion:**

A decrease in serum alkaline phosphatase levels, particularly when assessed as a percentage of age‐ and sex‐specific reference intervals, may be a valuable and noninvasive supportive feature of KFD in pediatric patients.

## Graphical abstract

The diagnosis of Kikuchi‐Fujimoto disease remains challenging and often requires invasive biopsy. Serum alkaline phosphatase levels were < 50% of the age‐ and sex‐specific median values in patients with Kikuchi‐Fujimoto's disease. Decreased serum alkaline phosphatase levels, mainly when assessed as a percentage of age‐ and sex‐specific reference intervals, may be a valuable and noninvasive diagnostic, supportive feature of Kikuchi‐Fujimoto disease in pediatric patients.

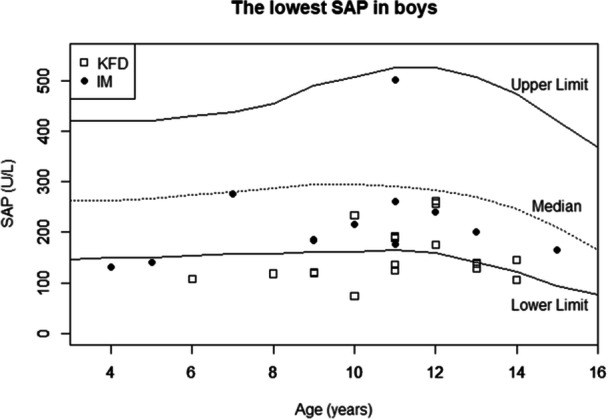

## 1 Introduction

Kikuchi‐Fujimoto disease (KFD), or histiocytic necrotizing lymphadenopathy, is characterized by prolonged fever and focal lymphadenopathy of the cervical lymph nodes [1]. It most often occurs in Asian women aged < 40 years and is uncommon in children [2]. The pathogenesis of KFD remains unclear; however, it is considered to be related to the immune response of T cells, histiocytes, and macrophages, as well as cytokine production against viral infections, such as human herpes virus six and Epstein–Barr virus [3]. Besides, KFD has been associated with chronic diseases such as systemic lupus erythematosus, VEXAS syndrome, and cryptogenic organizing pneumonia [4, 5, 6]. KFD poses diagnostic challenges owing to its resemblance to other infectious and noninfectious lymphadenopathies [2, 7]. The diagnosis is confirmed histologically via excisional biopsy of the affected lymph nodes. As KFD is a self‐limiting disease that improves within a few weeks to months without any treatment, the decision to perform a lymph node biopsy poses a clinical challenge due to its invasive nature [8, 9]. Administering systemic corticosteroids without a proper diagnosis may mask underlying conditions such as inflammatory diseases or hematological malignancies. Leukocytopenia, increased lactate dehydrogenase (LDH), and marginally increased C‐reactive protein levels contribute to diagnosing KFD; however, these findings are not highly specific [8, 10]. Thus, the development of simpler and more reliable diagnostic methods is imperative.

Serum alkaline phosphatase (SAP) is an enzyme involved in bone mineralization and hepatic homeostasis. SAP levels are considerably higher during childhood than during adulthood, especially during adolescence when skeletal growth and bone metabolism are accelerated [11]. Elevated SAP has been extensively investigated as a diagnostic hallmark of various pathological states, including osteogenic tumor‐related osseous metastases, cholestatic disorders, and hepatocellular impairment. Conversely, a low SAP activity occurs only in specific situations. Low SAP levels are frequently observed in patients with KFD and have been reported as a unique characteristic of this disease [12]. In addition, SAP can be measured in a straightforward and noninvasive manner. However, the previous study regarding SAP levels in patients with KFD was limited by the small sample size and absence of sex‐ and age‐specific SAP reference intervals.

This study aimed to investigate SAP levels in children with KFD. We hypothesized that SAP levels in pediatric patients with KFD would be lower than those in patients with other benign diseases characterized by prolonged fever. Identifying decreased SAP levels as a useful indicator in pediatric patients with KFD can aid in developing early and noninvasive diagnostic methods. Additionally, we investigated the relationship between decreased SAP levels and the clinical and laboratory findings in patients with KFD.

## 2 Methods

### 2.1 Study Design and Patient Selection

This retrospective observational study included patients aged < 16 years diagnosed with KFD or infectious mononucleosis (IM) and treated at the Department of Pediatrics of the NHO Okayama Medical Center between April 2016 and March 2023.

We selected IM as a comparison disease toward KFD because patients with IM share a similar age predilection and manifestations, such as prolonged fever, lymphadenopathies, cutaneous rash, hepatosplenomegaly, and a self‐limiting course.

**Figure 1:**
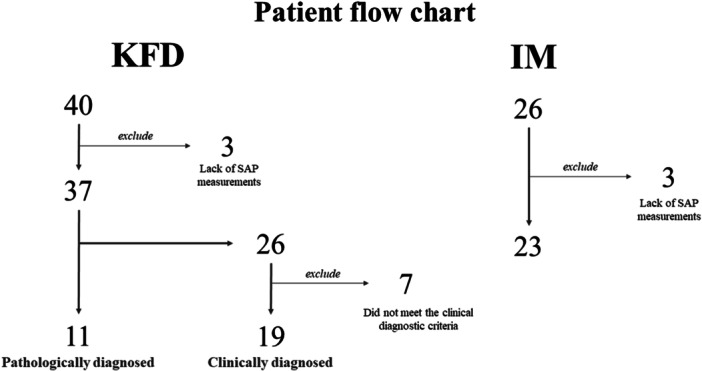
Flowchart of the participants.

A patient flowchart is shown in Figure 1. First, we performed complete consecutive enrollment and extracted all patients diagnosed with KFD or IM from electronic medical records during the study period, which included 40 patients with KFD and 26 patients with IM. Demographic, clinical, and laboratory data were extracted from the electronic medical records. The following exclusion criteria were established: patients who were receiving medications that affected bone turnover, had immune conditions, a history of bone fractures within the past year, a family history of hypophosphatasia, or underlying medical conditions that caused liver dysfunction, nutritional issues, or daily mobility impairments. However, none of the patients were excluded based on these criteria. We also excluded patients from either group who had no SAP measurements during the study period. Three patients were excluded from each group.

Of the 37 patients diagnosed with KFD, those who were pathologically or clinically diagnosed based on the following criteria. Pathological diagnosis was confirmed by lymph node biopsy. Clinical diagnosis was defined for patients who met all the following criteria [12]:

1. Prolonged fever (> 38.0°C) for over a week.
2. Unilateral or bilateral cervical lymphadenopathy with swelling and tenderness.
3. The nadir white blood cell count was < 4000/μL during the febrile period.
4. Spontaneous resolution without corticosteroid therapy.
5. Negative Epstein–Barr virus antibody titer (enzyme immunoassay) and antinuclear antibody.

Eleven patients were diagnosed pathologically and 26 were clinically diagnosed. Among the 26 clinically diagnosed cases, 7 were excluded owing to the absence of laboratory tests to rule out viral infection. Of the 23 patients diagnosed with IM, we included those with positive serological tests for Epstein–Barr virus or cytomegalovirus. The final analysis included 30 patients with KFD (19 with a clinical diagnosis and 11 with a pathological diagnosis) and 23 patients with IM.

### 2.2 Measurement of SAP

The lowest SAP level for each patient was recorded during the febrile period (body temperature > 38.0°C). SAP data before and after the febrile period were collected, when available.

The method used to measure SAP in Japan was modified in 2020. From April 2016 to November 2020, the SAP was measured by the Japan Society of Clinical Chemistry (JSCC) reference method. From December 2020 onward, SAP was measured by the International Federation of Clinical Chemistry (IFCC) reference method. SAP levels measured by the JSCC method were converted to IFCC reference values using the following formula [13]:

SAP(U/L)(IFCC method)=SAP(JFCC method)×0.35.

### 2.3 Evaluating SAP

The lowest SAP levels and other clinical characteristics were compared between the KFD and IM groups. SAP levels were also compared to the upper limit (97.5th percentile), median, and lower limit (2.5th percentile) of sex‐ and age‐specific reference intervals, as described previously [14] (details in Supporting Information S1: Table 1). SAP decline was defined as values < 2.5th percentile, based on the patient's age and sex. In addition, each patient's lowest SAP measurement during febrile periods was expressed as a percentage, applying an age‐ and sex‐specific median SAP level of 100%. We also compared the available SAP percentage values in patients with KFD at three distinct periods: before illness onset, at the minimum point during the febrile period, and after the fever subsided.

### 2.4 Statistical Analysis

Categorical data were presented as numbers and frequencies. They were compared using Pearson's chi‐square test or Fisher's exact test. Continuous variables are presented as median and interquartile range (IQR). These variables were compared using the Wilcoxon rank‐sum test. Statistical significance was defined as a two‐sided *p*‐value < 0.05. All statistical analyses were conducted using R (version 4.3.0 (2023‐04‐21 ucrt); Copyright I 2023 The R Foundation for Statistical Computing, Platform: x86_64‐w64‐mingw32/x64 (64‐bit).

### 2.5 Ethics Approval

This study was approved by our institution's Research Ethics Review Committee (registration number 2023‐004). Due to its retrospective nature, the requirement for informed consent from patients and/or their guardians was replaced by an opt‐out process. All procedures were performed in accordance with the Declaration of Helsinki and its later amendments.

## 3 Results

**Figure 2:**
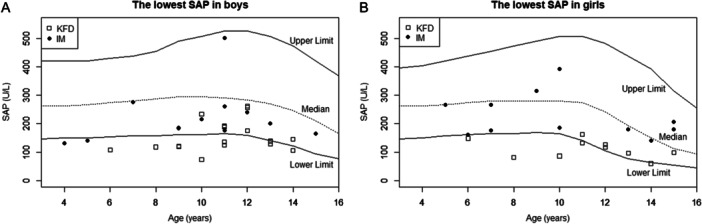
(A) Lowest SAP levels in boys with KFD or IM. (B) Lowest SAP levels in girls with KFD or IM. The scatter plot shows the lowest SAP levels in the KFD and IM groups. White squares and black circles indicate the lowest SAP levels in the patients with KFD and IM, respectively. The upper and lower solid lines indicate the upper (97.5th percentile) and lower (2.5th percentile) limits, respectively. IM, infectious mononucleosis; KFD, Kikuchi‐Fujimoto disease; SAP, serum alkaline phosphatase.

Table 1 shows the clinical and laboratory findings of the patients with KFD and IM. There were no significant differences in sex, age, date of minimum SAP, or fever duration between the KFD and IM groups. The lowest SAP level differed significantly between the two groups (KFD median, 131 IU/L; IQR, 110–159 IU/L; IM median, 206 IU/L; IQR, 176–267 IU/L). The lowest SAP levels in the KFD and IM groups were plotted according to age and sex (Figure 2A,B). Notably, 17 patients (56.7%) in the KFD group and two (8.7%) in the IM group showed a decline in SAP below the 2.5th percentile of the age‐ and sex‐specific reference intervals.

**Table 1:** Clinical and laboratory findings of patients with KFD and IM.

	KFD, *N* = 30^1^	IM, *N* = 23^1^	*p‐*value^2^
Boys/Girls	20/10	12/11	0.3
Age (years)	11.0 (10.0–12.8)	10.0 (7.0–12.5)	0.2
Lowest SAP (IU/L)	131 (110–159)	206 (176–267)	**< 0.001**
Date of the lowest SAP (days)	11.0 (8.0–14.8)	8.0 (7.0–11.0)	0.071
Duration of fever (days)	15.0 (11.0–19.3)	12.0 (10.0–14.0)	0.089
White blood cell count (/μL)	3000 (2300–3675)	7230 (5750–9500)	**< 0.001**
Neutrophil Count (/μL)	1203 (888–1604)	2890 (1513–3481)	**0.001**
Serum calcium (mmol/L)	2.23 (2.17–2.30)	2.27 (2.21–2.33)	0.2
Serum inorganic phosphorus (mmol/L)	1.39 (1.29–1.49)	1.29 (1.21–1.41)	0.074
Aspartate aminotransferase (IU/L)	44 (34–67)	86 (41–223)	**0.004**
Alanine aminotransferase (IU/L)	34 (21–70)	84 (31–298)	**0.008**
Albumin (g/L)	38 (35–41)	37 (34–42)	0.7
γ‐Glutamyl transpeptidase (μkat/L)	0.35 (0.25–0.52)	0.57 (0.25–0.97)	0.3
Lactate dehydrogenase (μkat/L)	7.95 (6.55–11.56)	8.32 (6.78–12.21)	0.6
C‐reactive protein (mg/L)	8.8 (2.4–19.7)	6.2 (4.0–13.4)	0.9

*Note:* Bold values denote statistically significant (*p* < 0.05).

Abbreviations: IM, infectious mononucleosis; IQR, interquartile range; KFD, Kikuchi‐Fujimoto disease; SAP, serum alkaline phosphatase.

^1^ *n* (%); Median (IQR).

^2^ Pearson's Chi‐squared test; Wilcoxon rank sum test.

**Figure 3:**
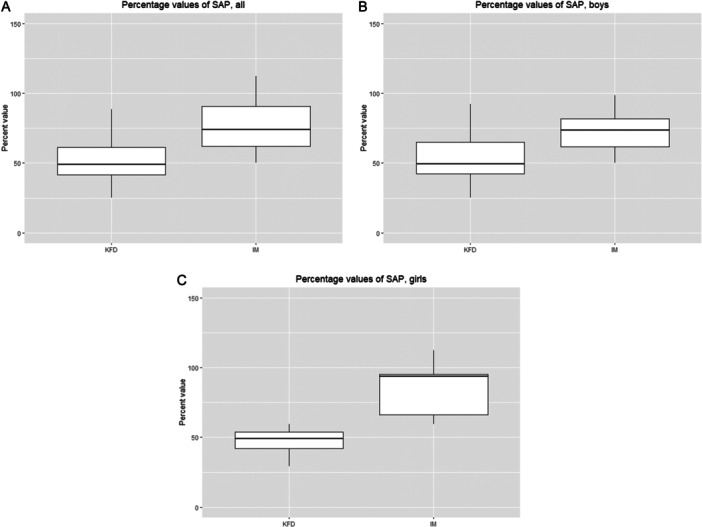
(A) Box‐and‐whisker plots of percentage SAP value. (B) Box‐and‐whisker plots of percentage SAP value in boys. (C) Box‐and‐whisker plots of rate SAP value in girls. The centerline denotes the median value (50th percentile), whereas the box contains the 25th and 75th percentiles of the data set. Black whiskers indicate the 5th and 95th percentiles. IM, infectious mononucleosis; KFD, Kikuchi‐Fujimoto disease; SAP, serum alkaline phosphatase.

The levels of serum calcium, inorganic phosphorus, albumin, and γ‐glutamyl transferase (γ‐GTP), which can affect SAP levels, did not differ between the KFD and IM groups. The lowest SAP levels in patients with KFD were ≤ 50% of the median SAP value for each specific age and sex category (Table 2, Figure 3A–C).

**Table 2:** Percentage of lowest SAP values of patients with KFD and IM.

	KFD^1^	IM^1^	*p*‐value^2^
Percentage value – All (% (IQR), *n*)	49.1 (41.6–61.2), *N* = 30	82.3 (62.3–99.5), *N* = 23	< 0.001
Percentage value – Boys (% (IQR), *n*)	49.4 (42.5–65.1), *N* = 20	74.0 (61.9–86.0), *N* = 12	0.005
Percentage value – Girls (% (IQR), *n*)	49.1 (42.0–53.7), *N* = 10	95.0 (74.2–126.1), *N* = 11	< 0.001

Abbreviations: IM, infectious mononucleosis; IQR, interquartile range; KFD, Kikuchi‐Fujimoto disease.

^1^ *n* (%); Median (IQR).

^2^ Wilcoxon rank sum test.

Most of the lowest SAP percentage values were observed during the febrile period in patients with KFD (details are outlined in Supporting Information S3: Figure).

Clinical and laboratory findings, including the date of lowest SAP, duration of fever, and the ratio of definitively diagnosed cases, were not significantly different between patients with KFD who had SAP decline and those without (Table 3). Additionally, among the 11 patients with pathologically diagnosed KFD, 7 (63.6%) showed SAP decline, which was comparable to the overall KFD patient cohort (56.7%). Furthermore, a comparison of patients with pathologically diagnosed KFD and IM showed similar results (Table 4). The lowest SAP levels differed significantly between groups (pathologically diagnosed KFD median, 129 IU/L; IQR, 123–151 IU/L; IM median, 206 U/L; IQR, 176–267 IU/L). Clinical and laboratory findings showed no significant differences between patients clinically diagnosed with KFD and those diagnosed pathologically (Supporting Information S2: Table 2). The percentage of SAP in patients with pathologically diagnosed KFD was also lower than that in patients with IM (Table 5).

**Table 3:** Clinical and laboratory findings of patients with KFD.

	SAP < 2.5th percentile, *N* = 17^1^	SAP ≧ 2.5th percentile, *N* = 13^1^	*p*‐value^2^
Boys/girls	12/5	8/5	0.7
Age (years)	11.0 (9.0–13.0)	12.0 (11.0–12.0)	0.071
Lowest SAP (IU/L)	121 (106–135)	175 (126–192)	**0.005**
Date of the lowest SAP (days)	11.0 (8.0–14.0)	11.0 (8.0–15.0)	0.7
Duration of fever (days)	15.0 (11.0–20.0)	14.0 (9.0–15.0)	0.2
Pathological/clinical diagnosis	7/10	4/9	0.7
White blood cell count (/μL)	3000 (2600–3300)	2600 (1700–3800)	0.6
Neutrophil count (/μL)	1320 (1044–1584)	910 (809–1835)	0.3
Serum calcium (mmol/L)	2.21 (2.16–2.30)	2.23 (2.20–2.30)	0.4
Serum inorganic phosphorus (mmol/L)	1.31 (1.15–1.49)	1.42 (1.36–1.45)	0.065
Aspartate aminotransferase (IU/L)	47 (38–68)	37 (23–62)	0.2
Alanine aminotransferase (IU/L)	34 (22–48)	56 (20–76)	> 0.9
Albumin (g/L)	36 (33–41)	39 (38–40)	0.4
γ‐Glutamyl transpeptidase (μkat/L)	0.30 (0.23–0.52)	0.33 (0.28–0.52)	0.5
Lactate dehydrogenase (μkat/L)	9.13 (7.16–11.6)	7.11 (5.58–9.40)	0.2
Erythrocyte sedimentation rate (mm/h)	46 (31–54)	39 (21–59)	0.8
Ferritin (μg/L)	414 (274–610)	184 (106–427)	0.065
C‐reactive protein (mg/L)	8.8 (2.7–27.3)	4.7 (2.0–19.2)	0.4

*Note:* Bold value denotes statistically significant (*p* < 0.05).

Abbreviations: IQR, interquartile range; KFD, Kikuchi‐Fujimoto disease; SAP, serum alkaline phosphatase.

^1^ *n* (%); Median (IQR).

^2^ Fisher's exact test; Wilcoxon rank sum test.

**Table 4:** Clinical and laboratory findings of patients with pathologically diagnosed KFD and IM.

	Pathologically diagnosed KFD, *N* = 11^1^	IM, *N* = 23^1^	*p‐*value^2^
Boys/girls	8/3	12/11	0.3
Age (years)	11.0 (10.5–12.5)	10.0 (7.0–12.5)	0.2
Lowest SAP (IU/L)	129 (123–151)	206 (176–267)	**< 0.001**
Date of the lowest SAP (days)	10.0 (7.5–12.0)	8.0 (7.0–11.0)	0.4
Duration of fever (days)	15.0 (10.5–15.5)	12.0 (10.0–14.0)	0.5
White blood cell count (/μL)	3000 (2300–3350)	7230 (5750–9500)	**< 0.001**
Neutrophil count (/μL)	1224 (923–1680)	2890 (1513–3481)	**0.019**
Serum calcium (mmol/L)	2.23 (2.19–2.29)	2.27 (2.21–2.33)	0.2
Serum inorganic phosphorus (mmol/L)	1.42 (1.26–1.44)	1.29 (1.21–1.41)	0.2
Aspartate aminotransferase (IU/L)	44 (27–57)	86 (41–223)	**0.008**
Alanine aminotransferase (IU/L)	34 (21–47)	84 (31–298)	**0.018**
Albumin (g/L)	39 (37–41)	37 (34–42)	0.7
γ‐Glutamyl transpeptidase (μkat/L)	0.32 (0.27–0.47)	0.57 (0.25–0.97)	0.4
Lactate dehydrogenase (μkat/L)	9.13 (6.96–11.0)	8.32 (6.78–12.21)	0.8
C‐reactive protein (mg/L)	3.7 (2.5–8.9)	6.2 (4.0–13.4)	0.5

*Note:* Bold values denote statistically significant (*p* < 0.05).

Abbreviations: IM, infectious mononucleosis; IQR, interquartile range; KFD, Kikuchi‐Fujimoto disease; SAP, serum alkaline phosphatase.

^1^ *n* (%); Median (IQR).

^2^ Pearson's Chi‐squared test; Wilcoxon rank sum test.

**Table 5:** Percentage of lowest SAP values of patients with pathologically diagnosed KFD and IM.

	Pathologically diagnosed KFD^1^	IM^1^	*p*‐value^2^
Percentage value – All (% (IQR), *n*)	47.9 (42.9–59.5), *N* = 11	82.3 (62.3–99.5), *N* = 23	0.001
Percentage value – Boys (% (IQR), *n*)	47.4 (43.0–65.4), *N* = 8	74.0 (61.9–86.0), *N* = 12	0.025
Percentage value – Girls (% (IQR), *n*)	52.3 (29.3–59.5), *N* = 3	95.0 (74.2–126.1), *N* = 11	0.011

Abbreviations: IM, infectious mononucleosis; IQR, interquartile range; KFD, Kikuchi‐Fujimoto disease.

^1^ *n* (%); Median (IQR).

^2^ Wilcoxon rank sum test.

## 4 Discussion

In the current study, the SAP levels of pediatric patients diagnosed with KFD were significantly lower than those of patients diagnosed with IM. Over half of the patients with KFD had SAP levels ≤ 50% of the corresponding median SAP levels according to their specific age and sex.

Elevated SAP levels have been reported in several diseases; however, studies on low SAP levels are limited. Nutritional deficiencies, hypothyroidism, hypoparathyroidism, achondroplasia, hypophosphatasia, or Wilson disease cause decreased SAP levels [15]. These conditions are not associated with fever and are relatively uncommon. The scarcity of low SAP levels highlights the importance of supportive diagnostic features of KFD. This study demonstrated that 56.7% of patients with KFD experienced a decline in SAP, which confirms the results of a previous report [12]. A primary strength of this study is the exploration of decreased SAP levels and other factors that may be related to SAP values. No significant differences were observed between pediatric patients with KFD and IM regarding fever duration, date of lowest SAP levels during the febrile period, or levels of serum albumin, calcium, or phosphorus. This suggests that decreased SAP levels may be a specific indicator of KFD, and not simply a consequence of prolonged fever. The data of patients with and without SAP decline were also compared. Inflammatory markers, including LDH, ferritin, and erythrocyte sedimentation rate, showed no significant differences between these patient groups, suggesting that there was no relationship between clinical findings and SAP decline.

Alkaline phosphatase (AP) is a glycoprotein enzyme that catalyzes the hydrolysis of phosphatase to inorganic phosphate [16]. This enzyme family comprises four different isoenzymes, of which tissue‐nonspecific SAP (TNSAP) is the main circulating variant [17]. Osteoblasts and hepatocytes are the primary producers of TNSAP and are essential for bone mineralization and liver function. In healthy adults, approximately half of AP arises from the liver, whereas the remaining half is derived from the skeleton [17]. In contrast, during childhood, growth plate chondrocytes contribute to AP synthesis, resulting in a marked AP composition. Previous investigations have indicated that bone AP constitutes approximately 70%–90% of the total AP content in children [18, 19]. These variants can be distinguished by directly measuring additional biliary enzymes such as γ‐GTP or bone AP [20]. In the present study, over half of the minimum SAP values were lower than half of the age‐ and sex‐specific median values in male and female pediatric patients. This decrease suggests that the lower SAP levels resulted from a reduction in TNSAP. Additionally, elevated aminotransferase levels in the IM group may have contributed to higher liver SAP levels. However, there was no significant difference in the γ‐GTP levels between the KFD and IM groups. Thus, a reduction in bone SAP may have contributed to the overall decrease in SAP in these patients.

Several studies have investigated KFD pathophysiology. Previous investigations focusing on serum inflammatory cytokines revealed increased levels of interferon‐γ, tumor necrosis factor‐α, and interleukin‐6 [21, 22, 23]. Recent reports have highlighted that amplified gene expression in the type I interferon pathway is a hallmark response in patients with KFD [24, 25]. In other studies, interferon‐α/β inhibits osteoblast progenitor proliferation and differentiation by inhibiting AP activity and downregulating BMP‐2 expression or exerting inhibitory effects on matrix mineralization by reducing the expression of COL1A, fibulin, and laminin [26, 27]. The overall mechanism and role of cytokine markers specific to KFD remain elusive; however, their potential effects on diminishing SAP levels can be speculated. In this study, lower SAP values were observed during fever, whereas higher SAP values were noted in the pre‐ and post‐febrile periods among most patients. Collectively, these findings suggest a plausible link between the clinical course of KFD and SAP.

### 4.1 Limitations

This study has several limitations. First, more than half of the KFD diagnoses included in this study were based on clinical evaluation, with only nine cases having histological diagnoses. Data from pathologically and clinically diagnosed patients were compared, revealing no significant differences in the findings. Second, our hospital serves as a secondary medical institution, primarily for patients referred by clinics. Selection bias, particularly in the IM group, cannot be ruled out. Third, the method used to measure SAP levels changed during the study period. The JFCC method is known for its heightened sensitivity to small intestinal AP, which may lead to subtle differences compared to the IFCC method [13]. The SAP levels derived using the JFCC method were converted to IFCC values to overcome this limitation.

## 5 Conclusion

In conclusion, more than half of the patients with KFD demonstrated decreased SAP levels below the 2.5th percentile of the sex and age reference intervals. SAP levels in pediatric patients with KFD were considerably lower than those in patients with IM. Although the exact mechanism of KFD in SAP remains unclear, low SAP levels may serve as a useful supportive feature for diagnosing KFD, potentially negating invasive surgical procedures.

## Author Contributions

Y.H. conceived and designed the study. S.F. collected and analyzed the data and drafted the manuscript. Y.H. and J.S. critically reviewed the manuscript and supervised the study.

## Ethics Statement

This study was approved by the Research Ethics Review Committee of our institution (registration number 2023‐004). Owing to the retrospective nature of the study, the requirement for informed consent from the patients' guardians was replaced by an opt‐out process. All the procedures were performed in accordance with the Declaration of Helsinki and its amendments.

## Consent

The authors have nothing to report.

## Conflicts of Interest

The authors declare no conflicts of interest.

## Supporting information

Supporting information.

Supporting information.

Supporting information.

## Data Availability

The data supporting this study's findings are available upon request from the corresponding author [Y.H.]. The data were not publicly available because of restrictions that could compromise the privacy of the research participants.
