# Supplementary material for: Serum Alkaline Phosphatase Levels in Pediatric Kikuchi‐Fujimoto Disease: A Retrospective Observational Analysis
Source: Immun Inflamm Dis. 2025 Jan 21;13(1):e70129. doi: 10.1002/iid3.70129 (PMC11748210; doi:10.1002/iid3.70129)
Supplement: Supplementary file 1 — Supporting information. [file IID3-13-e70129-s001.docx]

**Supplementary Table 1. Serum alkaline phosphatase reference values for boys and girls**

Abbreviations; SAP: serum alkaline phosphatase, IFCC: the International Federation of Clinical Chemistry

| **SAP reference for Boys (U/L) (IFCC)** | | |  | **SAP reference for Girls (U/L) (IFCC)** | | |  |
| --- | --- | --- | --- | --- | --- | --- | --- |
| Age | 2.5th percentile | Median | 97.5th percentile | Age | 2.5th percentile | Median | 97.5th percentile |
| 5 | 150.5 | 266 | 420 | 5 | 157.5 | 266 | 420 |
| 6 | 154 | 273 | 430.5 | 6 | 161 | 273 | 437.5 |
| 7 | 157.5 | 280 | 437.5 | 7 | 164.5 | 280 | 455 |
| 8 | 157.5 | 287 | 455 | 8 | 164.5 | 280 | 472.5 |
| 9 | 161 | 294 | 490 | 9 | 168 | 280 | 490 |
| 10 | 161 | 294 | 507.5 | 10 | 164.5 | 280 | 507.5 |
| 11 | 164.5 | 290.5 | 525 | 11 | 140 | 273 | 507.5 |
| 12 | 159.3 | 283.5 | 525 | 12 | 105 | 241.5 | 483 |
| 13 | 140 | 269.5 | 507.5 | 13 | 77 | 192.5 | 437.5 |
| 14 | 122.5 | 245 | 472.5 | 14 | 63 | 150.5 | 392 |
| 15 | 94.5 | 210 | 420 | 15 | 54.25 | 112 | 315 |
| 16 | 77 | 164.5 | 367.5 | 16 | 45.5 | 94.5 | 255.5 |
