# Supplementary material for: Serum Alkaline Phosphatase Levels in Pediatric Kikuchi‐Fujimoto Disease: A Retrospective Observational Analysis
Source: Immun Inflamm Dis. 2025 Jan 21;13(1):e70129. doi: 10.1002/iid3.70129 (PMC11748210; doi:10.1002/iid3.70129)
Supplement: Supplementary file 2 — Supporting information. [file IID3-13-e70129-s003.docx]

**Supplementary Table 2. The characteristics of the patients diagnosed with KFD clinically and those diagnosed pathologically.**

Abbreviations; KFD: Kikuchi-fujimoto disease, SAP: serum alkaline phosphatase

^1^n(%); Median (IQR: Interquartile range)

^2^ Fisher’s exact test; Wilcoxon rank sum test

|  | Clinically diagnosed KFD, N=19^1^ | Pathologically diagnosed KFD, N=11^1^ | *p*-value^2^ |
| --- | --- | --- | --- |
| Sex |  |  | 0.3 |
| Boys/Girls | 12/7 | 8/3 |  |
| Age (years) | 11.0 (10.0–12.5) | 11.0 (10.5–12.5) | > 0.9 |
| Lowest SAP (IU/L) | 132 (103–162) | 129 (123–151) | 0.6 |
| Date of the lowest SAP (days) | 12.0 (8.50–15.0) | 10.0 (7.5–12.0) | 0.2 |
| Duration of fever (days) | 15.0 (11.0–20.5) | 15.0 (10.5–15.5) | 0.3 |
| White blood cell count (/μL) | 3,000 (2,400–3,750) | 3,000 (2,300–3,350) | 0.7 |
| Neutrophil count (/μL) | 1,182 (855–1,589) | 1,224 (923–1,680) | 0.8 |
| Serum calcium (mmol/L) | 2.23 (2.18–2.32) | 2.23 (2.19–2.29) | 0.6 |
| Serum inorganic phosphate (mmol/L) | 1.37 (1.30–1.49) | 1.42 (1.26–1.44) | 0.8 |
| Aspartate aminotransferase (IU/L) | 44 (36–87) | 44 (27–57) | 0.4 |
| Alanine aminotransferase (IU/L) | 34 (21–90) | 34 (21–47) | 0.4 |
| γ-Glutamyl transpeptidase (μkat/L) | 0.33 (0.25–0.50) | 0.32 (0.27–0.47) | > 0.9 |
| Albumin (g/L) | 37 (34–40) | 39 (37–41) | 0.3 |
| Lactate dehydrogenase (μkat/L) | 7.70 (6.30–11.8) | 9.13 (6.96–11.0) | > 0.9 |
| C-reactive protein (mg/L) | 13.7 (3.4–23.3) | 3.7 (2.5–8.9) | 0.4 |
