# Supplementary figures and images for: Serum Alkaline Phosphatase Levels in Pediatric Kikuchi‐Fujimoto Disease: A Retrospective Observational Analysis
Source: Immun Inflamm Dis. 2025 Jan 21;13(1):e70129. doi: 10.1002/iid3.70129 (PMC11748210; doi:10.1002/iid3.70129)

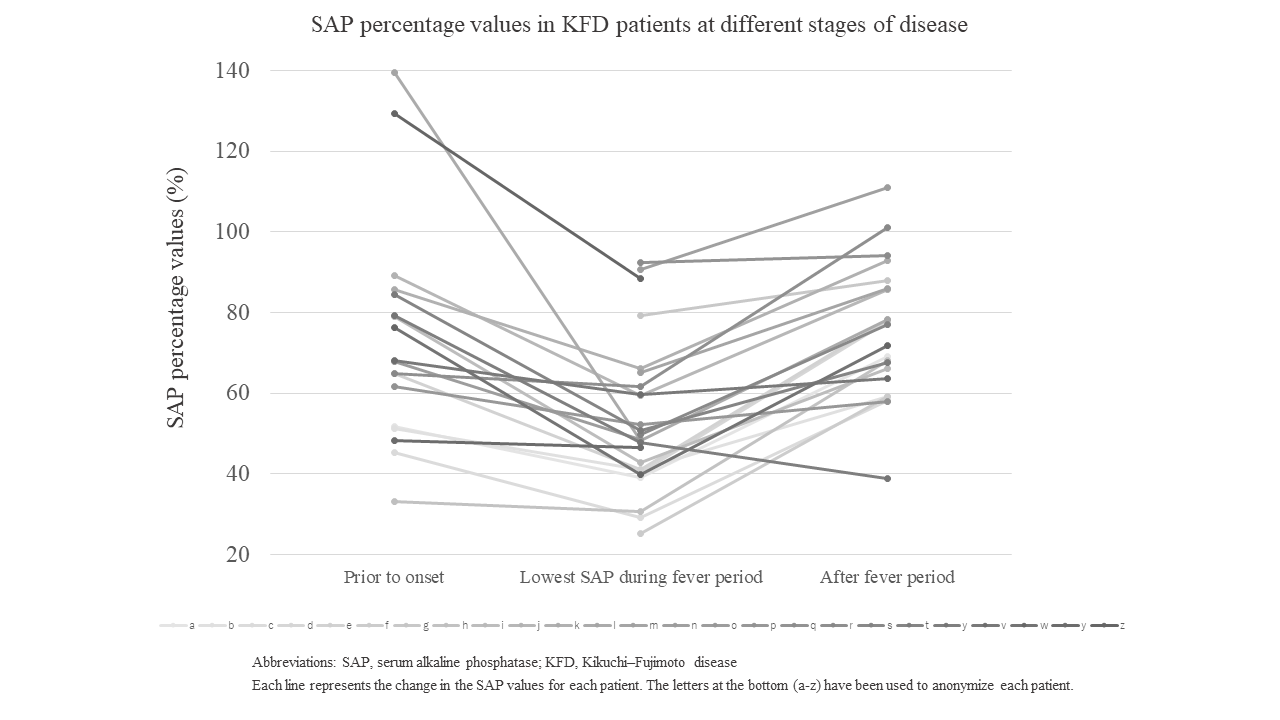

Supplement: Supplementary file 3 — Supporting information. [file IID3-13-e70129-s002.tif]
